# Molecular Insights into the Astringency of *Clitoria ternatea* Tea: Role of Phenolic Structure, Oral Constituents, and pH

**DOI:** 10.1021/acs.jafc.5c06189

**Published:** 2025-10-10

**Authors:** Inês E. Silva, Joana Vieira, Carlos Guerreiro, Joana Oliveira, Elsa Brandão, Victor de Freitas, Susana Soares

**Affiliations:** REQUIMTE, LAQV, Department of Chemistry and Biochemistry, Faculty of Sciences, 26706 University of Porto, Rua do Campo Alegre, s/n, Porto 4169-007, Portugal

**Keywords:** astringency, anthocyanins, flavonols, oral cells, oral cell-based model, salivary proteins

## Abstract

Astringency is a key sensory attribute in plant-based products, often linked to phenolic compounds, such as anthocyanins and flavonols. This study explores the molecular basis of astringency in butterfly pea flower tea using a cell-based model comprising human saliva, oral mucosal pellicle, and HSC3 oral epithelial cells. Specifically, it aimed to (i) assess the contribution of oral constituents to phenolic compound binding (ternatins, flavonols); (ii) evaluate compound-specific interactions; and (iii) determine the influence of pH. Significant interactions with HSC3 oral cells were observed, though inhibited by salivary proteins. Structural features of phenolic compounds also modulated interactions: *p*-coumaroyl residues enhanced, while rhamnosyl residues reduced them. Acidic conditions promoted binding through the neutral quinoidal base of ternatins interacting with epithelial membranes, whereas at neutral pH, anionic forms were repelled by negatively charged oral cells. These findings clarify how phenolic structure, oral constituents, and pH govern oral interactions, providing molecular insight into astringency perception.

## Graphical abstract

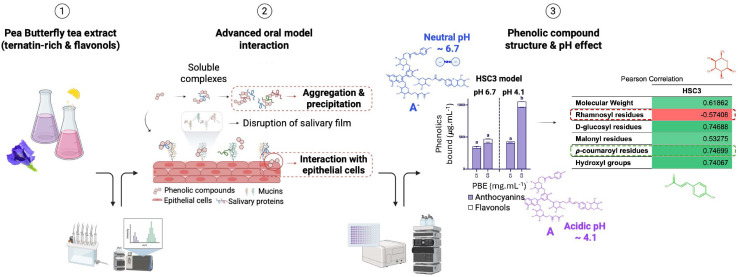

## Introduction

Astringency is perceived as a tactile and diffuse sensation characterized by roughness, puckering, and tightening in the mouth.^1–3^ The molecular mechanisms underlying astringency and its perception have been extensively discussed.^4–11^ The prevailing mechanism, proposed by Bate–Smith in 1954, is based on the interaction and precipitation of salivary proteins by phenolic compounds, leading to the formation of salivary complexes.^12–14^ Additional hypotheses suggest that astringency may also result from the binding of phenolic compounds to oral epithelial cells^15^ and/or the activation of mechanoreceptors.^16,17^ Furthermore, astringency perception varies between individuals and is influenced by multiple factors, including the concentration of astringent compounds and the pH of the medium (acidity).^18^

In plant-based foods, astringency is typically associated with tannins, a major class of phenolic compounds.^18^ However, other compounds such as flavonols and anthocyanins have also been mentioned to cause astringency.^19^ Flavonols are widely distributed in edible plants, vegetables, and fruits. Some flavonol glycosides have been associated with astringency, particularly at low detection threshold, and contribute to subqualities such as velvety, silky, and coating sensations. Interestingly, although certain flavonols are perceived as astringent, they fail to promote the aggregation and precipitation of salivary proteins, suggesting the involvement of alternative mechanisms. Guerreiro et al. reported that quercetin and its derivatives primarily interact with oral epithelial cells (e.g., tongue cell lines) rather than salivary proteins, indicating a potential alternative pathway for astringency perception.^20^

Anthocyanins are well known for their wide color spectrum and pH dependency.^21,22^ Although they are not typically associated with astringency, several studies have reported their (in)­direct contribution to modulate astringency perception.^19,23–26^ For instance, Gonzalo-Diago et al. and Paissoni et al. showed that wine fractions rich in acetylated and coumaroylated anthocyanins seem to enhanced perceptions of astringency and bitterness.^24,25^ Although common anthocyanins are generally described as having a very “mild taste”,^27^ their addition to grape seed and skin extracts has been found to intensify astringency,^28^ in particular subqualities as “fine grain”, along with other “in-mouth” sensations, like “dry”, “grippy”.^23^ Similar findings have been reported in model wine systems, where anthocyanins contributed to descriptors such as “fullness”, “chalkiness”, and “coarseness” sensation,^29^ as well as increased “dryness” and “roughness”.^30^ The mechanisms behind these sensory effects are under investigation. Some studies suggest that monoglucoside,^29,31^ acetylated, and coumaroylated anthocyanins can interact with salivary proteins to varying extents, with the latter forms showing the highest reactivity.^26^ More recently, other oral components beyond salivary proteins have been suggested to play a role in anthocyanin-induced astringency.^20^ Despite emerging evidence, the exact mechanisms and structure–activity relationships responsible for these interactions remain unclear.

To unravel the mechanisms of anthocyanin-induced astringency, an interesting case study is *Clitoria ternatea* L. tea, commonly known as “Butterfly pea” tea.^32^ In food applications, *C. ternatea* blue extract is often used as a natural food coloring^33^ and as an herbal beverage, resembling the flavor of black tea and coffee,^34–36^ and inducing astringency. To improve the flavor profile of butterfly pea tea and reduce its astringency, the traditional addition of a slice of lemon is commonly practiced.^35^ This not only enhances its visual appeal by inducing a color change but also leads to a more palatable flavor profile, partly by lowering the pH, which can influence the chemical form and possible sensory perception of anthocyanins. To date, it has been described that petals from *C. ternatea* contain many phytochemical compounds, namely 14 flavonols^37,38^ and 15 (poly)­acylated anthocyanins commonly known as ternatins.^33,39–42^ While most astringency studies focus on the interaction between individual compounds and salivary proteins, they often overlook the complexity of the oral environment, where different components can interact dynamically, including salivary proteins, oral (cells) mucosa, and the oral mucosal pellicle.^43^

Therefore, this study was designed to provide insights into the following objectives: (i) to study the contribution of different oral constituents to the overall binding of the phenolic compounds of butterfly pea tea; (ii) to compare, both qualitatively and quantitatively, how the different phenolic compounds bind to the different oral constituents; and (iii) to assess the impact of pH (close to neutrality and acidic) on the previous binding interactions. To reach these objectives the study was conducted under a cell-based model incorporating the main oral constituents involved in astringency perception: human saliva, mucosal pellicle, and a tongue-derived oral cell line (HSC3).^31,44^

## Experimental Section

### Chemicals and Reagents

All reagents used were sourced from analytical grade. Acetonitrile (99.8%), ammonium persulfate (99.9%), and formic acid (99.0%) were acquired from Chem-Lab (Zedelgem, Belgium). The Pierce BCA Protein Assay Kit was purchased from Thermo Fisher Scientific (Massachusetts, MA, US). The bovine serum albumin (BSA) (≥96%), Dithiothreitol (DTT) (99%), mucin porcine stomach type II, sodium dodecyl sulfate (SDS), sodium carbonate, *N*,*N*,*N*′,*N*′-tetramethylethylenediamine (TEMED), trichloroacetic acid (TCA) (99.0%), trifluoroacetic acid (TFA) (99.0%), and trypan blue dye were obtained from Sigma-Aldrich (Darmstadt, Germany). Acetone, ethanol (96%), and sodium hydroxide (NaOH) were from LabChem (PA, US), methanol (99.8%) was acquired from Riedel-de Han (Seelze, Germany), hydrochloric acid was purchased from Fluka (Seelze, Germany), acetic acid from CARLO ERBA Reagents (Emmendingen, Germany), and urea from José M. Vaz Pereira, S.A. (Santarém, Portugal). The kaempferol-3-*O*-rutinoside, quercetin-3-*O*-rutinoside, and their respective aglycone standards were purchased from Extrasynthese (Lyon, France). Acrylamide/Bis (40%), Tris-base, Tris-Glycine Buffer (99.9%), and SDS-page sample loading buffer (5×) were acquired from NzyTech (Lisbon, Portugal). The Milli-Q EQ 7000 ultrapure water purification system was from Merck Millipore (Massachusetts, MA, US) with a Millipak 0.22 μm hydrophilic membrane filter. The dimethyl sulfoxide (DMSO) (99.5%, anhydrous) was purchased from Scharlau (Barcelona, Spain).

### Phenolic Compounds Purification from a Commercial *C. ternatea* Extract

In this study, a commercial extract of *C. ternatea* from Zhejiang Binmei Biotechnology CO., LTD (Zhejiang, China) (Batch No: BFE220726) (hereafter referred to as a commercial extract) was used as the raw material. Initially, proteins were eliminated by applying the TCA protocol.^45^ Specifically, 2.240 mL of a 25% TCA solution in acetone was added to 500 mg of a commercial extract previously diluted in 5 mL of deionized water. Subsequently, the solution was transferred to 1.5 mL microtubes and subjected to a temperature of −20 °C for 45 min, followed by a centrifugation at 8000 *g* for 10 min at 4 °C. To eliminate polysaccharides and other potential interferences, the resulting supernatants were subjected to a solid-phase extraction step using an Oasis HLB C18 35 cc Vac cartridge (Waters, Massachusetts, US). The activation and conditioning of the cartridge were initiated with 2 column volumes of methanol and washed with 5 column volumes of deionized water. The diluted supernatants were loaded into the cartridge. The stationary phase was washed with 5 column volumes of deionized water, and the phenolic compounds were eluted from the cartridge with a methanol/acetonitrile mixture (90:10, v/v), followed by a methanol solution acidified with 2% HCl. The resultant organic solvent was removed from the solution by using a rotary evaporator. The resulting butterfly pea extract was then lyophilized and stored at −20 °C until high-performance liquid chromatography (HPLC) analysis (detailed below), yielding an overall composition of 94% anthocyanins and 6% flavonols. In subsequent sections, this extract will be termed the “pea butterfly extract” (PBE).

### *C. ternatea* Phenolic Compounds-Rich Extract Characterization

To identify the compounds within the PBE, 50 μL were analyzed using liquid chromatography–mass spectrometry (LC-MS), using a Finnigan DECA XP PLUS instrument (Massachusetts, US) equipped with an Agilent Poroshell 120, C18 reverse-phase column (250 × 4.6 mm, 2.7 μm particle diameter) (California, US). The chromatographic separation used two solvents: solvent A, consisting of 1% formic acid in water, and solvent B, consisting of 1% formic acid in 30% acetonitrile. The gradient elution program was initiated with 35% solvent B and gradually increased to 80% solvent B at 65 min, reaching 100% at 66 min, and maintaining this composition until 76 min. At 78 min, the gradient returned to its initial conditions after 85 min. The flow rate was set at 0.4 mL · min^–1^ with detection at 280, 340, and 540 nm.^40^

For the determination of the total phenolic compound content of the extract, an HPLC system, Thermo Scientific Vanquish – Photodiode array detector (VH-D10-A) (Massachusetts, US), was used. The same column and LC method mentioned above were employed for this analysis. Before HPLC analysis, all of the supernatants were acidified with 2% HCl. For flavonol quantification, kaempferol-3-*O*-rutinoside standard was used to obtain a calibration curve between 5.0 and 50.0 mg·L^–1^ (*y* = 14343.33*x* – 13.4867, *R* ^2^ = 0.98). For anthocyanin quantification, purified ternatin D1 was used to obtain a calibration curve between 25 and 300 mg·L^–1^ (*y* = 283.96*x* + 5.2906, *R* ^2^ = 0.99).

### Saliva Collection and Analysis

Unstimulated human saliva was isolated from a group of healthy male and female nonsmoker volunteers (10–15 volunteers) aged between 23 and 45 years. These volunteers were not taking any medications at the time of the study. Saliva collection was conducted between 1:30 and 2:30 pm. Participants were instructed to refrain from ingesting any food or beverage for at least 1 h before saliva collection. Following this, volunteers accumulated unstimulated saliva for 10 min and spat on a tube. Then, the saliva samples were combined, creating a representative pool of samples of human saliva. To stabilize human saliva, a 10% solution of TFA (final concentration of 0.1%) was used to inhibit protease activity and remove high-molecular-weight protein. The saliva was then subjected to centrifugation at 8000 *g* for 5 min at 4 °C. The resulting supernatant was recovered, and its physical-chemical parameters were characterized, including ionic strength, protein concentration, pH, and salivary protein profile. Ionic strength was determined to be 5220 μS·cm^–1^ by using a conductometer (WTM Inolab 740, Oberbayern, Germany). Protein concentration was determined to be 1421 μg·mL^–1^ by bicinchoninic acid assay (The Pierce BCA Protein Assay Kit), a value consistent with the typical range reported for healthy human saliva (500–2000 μg·mL^–1^).^46^ Saliva pH was measured using a METTLER TOLEDO (Greifensee, Switzerland) InLab Semi-Micro pH electrode, indicating a pH of 2.56. Following these evaluations, the salivary protein profile was analyzed by HPLC. Saliva was injected onto a Jasco LC-4000 system with a photodiode array detector (MD-4010) (Tokyo, Japan) equipped with a Kinesis Telos reversed-phase C8 column (Altrincham, UK), with a 5 μm diameter and dimensions of 150 × 2.1 mm. The chromatographic separation used two solvents: solvent A, consisting of 0.2% TFA in water, and solvent B, consisting of 0.2% TFA in an acetonitrile/water mixture (80:20, v/v). The gradient elution program was initiated with 15% solvent B and gradually increased to 46% solvent B at 35 min, reaching 55% at 40 min and maintaining this composition until 44 min. At 45 min, the gradient increased to 90% solvent B, and then to 100% solvent B until 55 min, maintaining this composition until 57 min. Subsequently, it returned to its initial gradient after 67 min. The flow rate was set at 0.5 mL·min^–1^ with detection at 214 nm.^44^ Before application in the cell-based oral model, saliva underwent a 1.5× concentration through lyophilization to replace water by HANK’s buffer (Sigma-Aldrich).

An informed consent form was signed by each participant before the saliva isolation. This study was conducted according to the declaration of Helsinki and was approved by the Ethics Committee of Faculty of Sciences (ref: CE2022/p40; 28/02/2023).

### Cell Culture Conditions

In this study, the HSC3 (JCRB0623-A; Tebubio, Lisbon, Portugal) cell line obtained from the oral epithelium of the human tongue was used. The oral epithelial cell growth was achieved using a culture medium of DMEM-high glucose supplemented with 1% Ala-Gln (200 mM solution) from Sigma-Aldrich. To guarantee the optimal growth conditions for HSC3 cells, the culture medium was fortified with 10% premium heat-inactivated Fetal Bovine Serum (Biowest, Nuaillé, France) and 1% of an antibiotic/antimycotic solution containing 100 U·mL^–1^ of penicillin, 100 μg·mL^–1^ of streptomycin, and 0.25 μg·mL^–1^ of amphotericin B, all from Sigma-Aldrich. The cells were maintained in a controlled environment with 5% CO_2_ in air at a temperature of 37 °C within a humidified atmosphere. Subculturing was performed in a Telstar Microbiological Safety Cabinet Bio II Advance Plus (Barcelona, Spain) when the cells reached 90–95% confluence, using 0.25% (w/v) trypsin-EDTA_4_Na for cell dissociation.

### Interaction of the PBE with the Oral Model

The 2D oral model employed in this study had been previously described by Soares et al. (2019),^44^ which mimics key components of the human oral cavity involved in astringency perception (salivary proteins, oral (cells) mucosa, and the oral mucosal pellicle). Two pH conditions were evaluated to reflect real-life beverage consumption: pH 6.0, corresponding to a typical beverage pH, and pH 3.0, determined through an in-house preparation of butterfly pea tea with lemon addition, reflecting traditional consumption practices aimed at reducing astringency.

The HSC3 oral epithelial cells were seeded into 48-well flat-bottomed tissue culture plates at a density of 1.43 × 10^5^ cells/well. They were allowed to grow for 72 h until they reached 90–100% confluence, at which point they were ready to be used. To assess the contribution of different oral constituents to the overall binding of phenolic compounds from PBE, the 48-well plates were divided into 4 experimental conditions: (i) HSC3 + Mu + SP, (ii) HSC3 + Mu, (iii) HSC3 + SP, and (iv) HSC3. A schematic representation of the experimental setup is shown in Figure 1.

**Figure 1:**
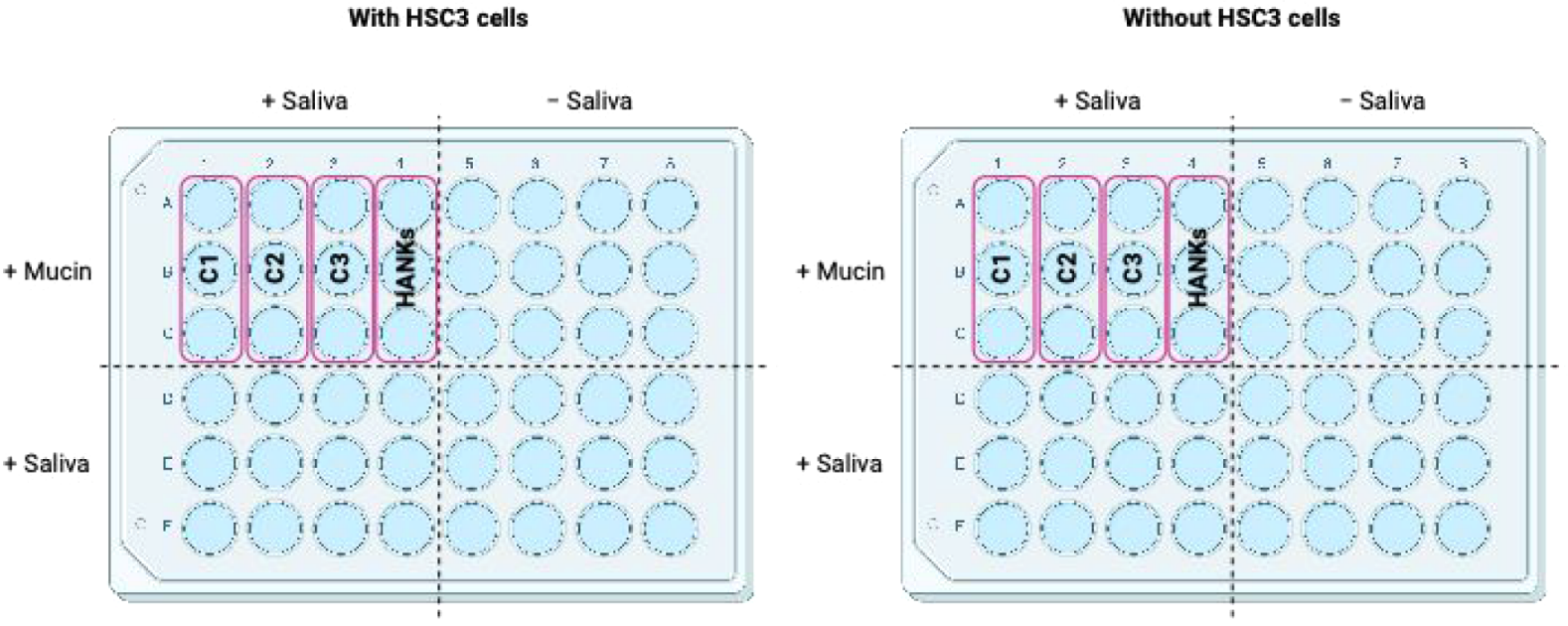
Schematic representation of the experimental setup, both with and without the HSC3 oral epithelial cell. C1, C2, and C3 represent the increasing concentrations tested in the assay (C1 < C2 < C3), with HANK’s buffer serving as the control.

Before the mucosal pellicle was formed, the growth medium was removed from 24 of the 48-well plates by washing the cell monolayers twice with warm (at 37 °C) phosphate-buffered saline (PBS) (Sigma-Aldrich). A stock solution of 20 mg·mL^–1^ porcine stomach type II mucin was prepared in 1 M NaOH and then diluted with HANK’s buffer to achieve a final concentration of 1.0 mg·mL^–1^. After the pH was adjusted to 7.0, 75 μL of this solution was added to 24 wells according to the plate layout. While the 1 h incubation occurred under the same conditions as cell growth, a separate 48-well plate without cells was prepared with the same layout as that with the cells. Postincubation, the mucin solution was carefully aspirated from the wells, and 75 μL of prewarmed (37 °C) saliva at a concentration of 800 μg·mL^–1^, with pH adjusted to 7.0, was added to 12 of the 24 wells (HSC3 + Mu + SP) and incubated for 2 h. For the other 12 wells, HANK’s buffer was added instead of saliva (HSC3 + Mu). At the same time, the 12 wells without mucin were washed twice with warm PBS and saliva was also added as described before (HSC3 + SP). In the remaining 12 wells, HANK’s solution was added instead of saliva and allowed to incubate for 2 h (HSC3). In the end, 12 wells of each condition were set up in the cell plate (HSC3 + Mu + SP, HSC3 + Mu, HSC3 + SP, and HSC3) and in the plate without cells (Mu + SP, Mu, SP, HANK’s). After the incubation period, saliva was carefully aspirated from saliva-containing wells and replaced with prewarmed fresh human saliva. Finally, 75 μL of three different stock solutions of PBE (2.66, 5.32, and 11.97 mg·mL^–1^) previously adjusted to pH 6.0 or pH 3.0 and previously warmed (at 37 °C), were added to their respective wells. This resulted in final concentrations of 1.33, 2.66, and 5.99 mg·mL^–1^, which are mentioned throughout the manuscript. These mixtures were incubated for 15 min under the same conditions used for cell growth. In some wells, HANK’s solution (at pH 6.0 or pH 3.0) was added instead of the PBE to control cell viability and saliva profile. The final pH of the interactions was measured. For samples initially adjusted to pH 3.0, the final pH ranged from 3.8 to 4.2 and henceforth is reported as pH 4.1. For samples initially adjusted to pH 6.0, the final pH ranged from 6.5 to 6.9 and is henceforth reported as pH 6.7.

After incubation, the solutions were collected to microtubes, kept in ice, and centrifuged at 8000 *g* for 5 min. The resulting supernatants were immediately frozen at −80 °C until further analysis.

### In-Plate Measurement of the Phenolic Compounds Bound to a Cell-Based Oral Model

To investigate the interaction between the PBE and the oral model, the same two 48-well microplates used in the previous assay were repurposed for microplate analysis. To ensure the complete removal of any residual solutions, the wells were carefully aspirated, followed by the addition of 90 μL of DMSO previously acidified with 5 μL of 2% HCl into each well. A subsequent 15 min incubation period in the dark at room temperature was performed. After this incubation, 80 μL of the contents from each well were transferred to a 96-well microplate, and the absorbance at 280, 360, and 540 nm was determined using a UV–vis microplate reader (Biotek4 PowerWave XS, New England, VT, USA).

For quantifying the total concentration of compounds bound to the oral model, the isolated ternatin D1 and commercial kaempferol-3-*O*-rutinoside were used to specifically quantify phenolic compounds from the anthocyanin and flavonol classes, respectively. The calibration curves covered a concentration range of 0–0.2 mg·mL^–1^. For ternatin D1, the equation was determined to be *y* = 0.28660*x* + 0.0006 (*R* ^2^ = 0.99), while for kaempferol-3-*O*-rutinoside, it was *y* = 4.95*x* + 0.0089 (*R* ^2^ = 0.99).

### HPLC Analysis of the Interactions of the PBE with the Oral Model

The supernatants obtained from the experimental conditions exhibiting the most pronounced interactions by in-plate detection were subsequently analyzed using the same HPLC system, column, and solvent gradient mentioned above. Once again, prior to HPLC analysis, all of the supernatants were acidified with 2% HCl, and 50 μL of each sample was injected. This analytical approach allowed us to determine the changes in the phenolic compound profile. To quantify the concentrations of these compounds, the same calibration curves of commercial kaempferol-3-*O*-rutinoside and isolated ternatin D1 previously used for HPLC quantification of *C. ternatea* phenolic compounds were used, namely *y* = 283.96*x* + 5.2906, *R* ^2^ = 0.99 for anthocyanins and *y* = 14343.33*x* – 13.4867, *R* ^2^ = 0.98 for flavonols. The concentration of the bound phenolic compounds (expressed in μg·mL^–1^) retained within each model was determined according to the following expression:

[Phenolic compound bound] (μg·mL^–1^) = [phenolic compound in solution before incubation] – [phenolic compound in solution after incubation].

### Analysis of the Salivary Protein Profile Changes upon Interaction of the PBE with the Oral Model

To investigate the salivary proteins (eventually) precipitated during the interaction of the PBE with the complete oral model, the supernatants were analyzed by sodium dodecyl sulfate-polyacrylamide gel electrophoresis (SDS-PAGE). For the SDS-PAGE analysis, a 16% Acrylamide/Bis separating gel and a 4% Acrylamide/Bis stacking gel were used. The electrophoresis process was conducted on a Bio-Rad PowerPac Basic Power Supply (California, US) with 1× commercial Tris-Glycine Buffer containing 0.1% SDS as a cathode and anode buffer, following the Laemmli buffer protocol.^47^

For each condition, three replicates were carried out. In each case, 20 μL of the supernatants were diluted with 5× electrophoresis sample buffer, resulting in a final volume of 25 μL. Sample buffer was prepared by combining 5× commercial SDS-page sample loading buffer (NZYTech) with 9.5 M urea and 32 mM DTT. Subsequently, sample denaturation was performed by heating to 95 °C using a thermal mixer (Thermo Fisher Scientific) for 10 min while continuously shaking. Upon cooling, 20 μL of each sample was loaded into individual wells. The electrophoresis began at a voltage of 50 V for 30 min, and then the voltage was raised to 120 V until the dye front migrated completely out of the running gel. Afterward, gels were fixed with 40% methanol and 10% acetic acid for 1 h, after which they were stained with a Bio-Rad Coomassie Blue R-250 dye-based reagent (California, US) for 15 min. The destaining step involved sequential washes with water, followed by a destaining solution (20% methanol and 10% acetic acid) until the protein bands became visible. Protein molecular weights (11 to 245 kDa) were estimated by comparison with the marker NZY Color Protein Marker II from NZYTech.

### Cell Viability Assay

Cell viability was assessed by neutral red assay.^48^ After the removal of solutions from the wells, a 90 μL aliquot of a neutral red solution (Sigma-Aldrich) previously diluted in the culture medium (0.08 mg·mL^–1^) and prewarmed (at 37 °C) was added to each well. The plates were then incubated for 2 h under normal growth conditions. After the incubation period, the content of each well was carefully aspirated, and the wells were washed with 90 μL of PBS, pH 7.6. Finally, 90 μL of a working solution (50% ethanol/1% acetic acid) was added to each well, and the absorbance was measured at 540 nm using the same UV–vis microplate reader previously mentioned. Cell viability was determined and found to be between 77% and 87% with no significant differences in comparison to the control (Figure S1).

### Statistical Analysis

All experiments were conducted in three separate sets of triplicate on different days, with replicates included within the same plate assay. To assess the normality of the data, the Shapiro–Wilk test was performed. For data that exhibited a normal distribution, the mean values and standard error of the mean (SEM) were calculated and analyzed using 2-way analysis of variance (ANOVA). Subsequently, Tukey’s multiple comparison test was employed to discern significant differences. In contrast, data that did not conform to normality were subjected to the Kruskal–Wallis test, and the mean rank values were compared using the Uncorrected Dunn’s test. Statistical analyses were carried out using GraphPad Prism version 8.0 for Windows (California, US; www.graphpad.com). Principal component analysis (PCA) and Pearson analysis were conducted using OriginPro version 2024b for Microsoft Windows (Massachusetts, US; originlab.com). Supplementary data analysis was performed using Microsoft Excel (Washington, US; www.microsoft.com).

## Results and Discussion

### PBE Characterization

The obtained PBE was chemically characterized, revealing it to be a mixture of flavonoids, specifically, (poly)­acylated anthocyanins and flavonols. LC-MS data are detailed in Figure 2A, which shows the identified compounds, their retention times, UV signals, the ion masses, and the ion mass fragments (MS^2^). As a result, 18 phenolic compounds, 13 (poly)­acylated anthocyanins, and six flavonols were identified. The anthocyanins group, represented by peaks 1–3, 5, 7, 9, 12–15, and 17–18, included derivatives of delphinidin-3-glucoside and ternatins, while the flavonols, represented by peaks 4, 6, 8, 10–11, and 16 were identified as kaempferol and quercetin derivatives. Most of these identifications were based on the order of elution, spectral characteristics, and MS/MS fragmentation data through comparison with data available in the literature.^49–51^

**Figure 2:**
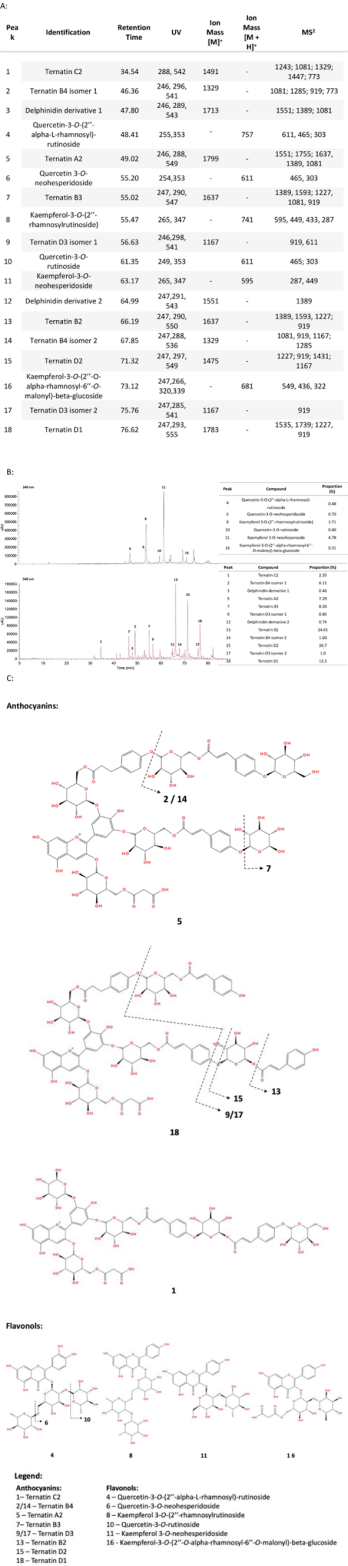
Identification and structure of phenolic compounds from PBE. Identifications were based on the LC-MS data (A) and the HPLC profile (flavonols detected at 340 nm and anthocyanins detected at 540 nm) (B) of the PBE at 0.665 mg·mL^–1^. The tables in (B) present the identity and relative proportion of each peak identified in the PBE based on the chromatographic area.

Additionally, Figure 2B shows the HPLC profile of the PBE at wavelengths of 340 and 540 nm. An assessment of the area under the chromatogram peaks revealed that, within the anthocyanin group, ternatin B2 (peak 13) and ternatin D2 (peak 15) were the main anthocyanins in the extract, while the delphinidin derivatives (peaks 3 and 12) exhibited the lowest abundance.^51^ In the case of flavonols, the most abundant compounds were kaempferol-3-*O*-neosheperoside (peak 11) and kaempferol-3-*O*-(2″-rhamnosylrutinoside) (peak 8), while quercetin-3-*O*-rutinoside (peak 10) and kaempferol-3-*O*-(2″-*O*-alpha-rhamnosyl-6″-*O*-malonyl)-beta-glucoside (peak 16) were among the least abundant. This is in agreement with the literature.^38^ The molecular structures of the anthocyanins and flavonols from PBE are presented in Figure 2C.

### Interaction of the PBE with the Oral Models

Although some studies have described oral cell interactions with different phenolic compounds, there remains a significant gap in knowledge regarding oral cell interactions with anthocyanins. In this study, an extract of butterfly pea flower, known for its abundance of (poly)­acylated anthocyanins and flavonols and previously described as an astringent, was used to study the ability of its phenolic compounds to bind to different oral constituents.

Figure 3 illustrates the general outcomes of the interaction between different concentrations of the PBE to simulate the tea brewing process (1.33, 2.66, and 5.99 mg·mL^–1^) at two pH values (pH 4.1 and pH 6.7) and the oral models under investigation (monolayer of the HSC3 cell line, HSC3 cells incubated with human saliva (HSC3 + SP), HSC3 cells preincubated with mucin (HSC3 + Mu), and HSC3 cells with both mucosal pellicle and saliva (HSC3 + Mu + SP)). This astringent tea is served alongside a slice of lemon to give it a milder astringency sensation,^35,52^ and given the well-known sensitivity of anthocyanins to pH, it was decided to study the effect of pH on the binding of PBE phenolic compounds toward the oral constituents. After the interaction, the bound phenolic compounds were quantified by direct measurements of the absorption at specific wavelengths (540 nm for anthocyanins and 340 nm for flavonols) (see section In-Plate Measurement of the Phenolic Compounds Bound to Cell-Based Oral Model from the Experimental Section). The interaction results were quantified using ternatin D1 equivalents (μg·mL^–1^) for the anthocyanins and kaempferol-3-*O*-rutinoside equivalents (μg·mL^–1^) for the flavonols.

**Figure 3:**
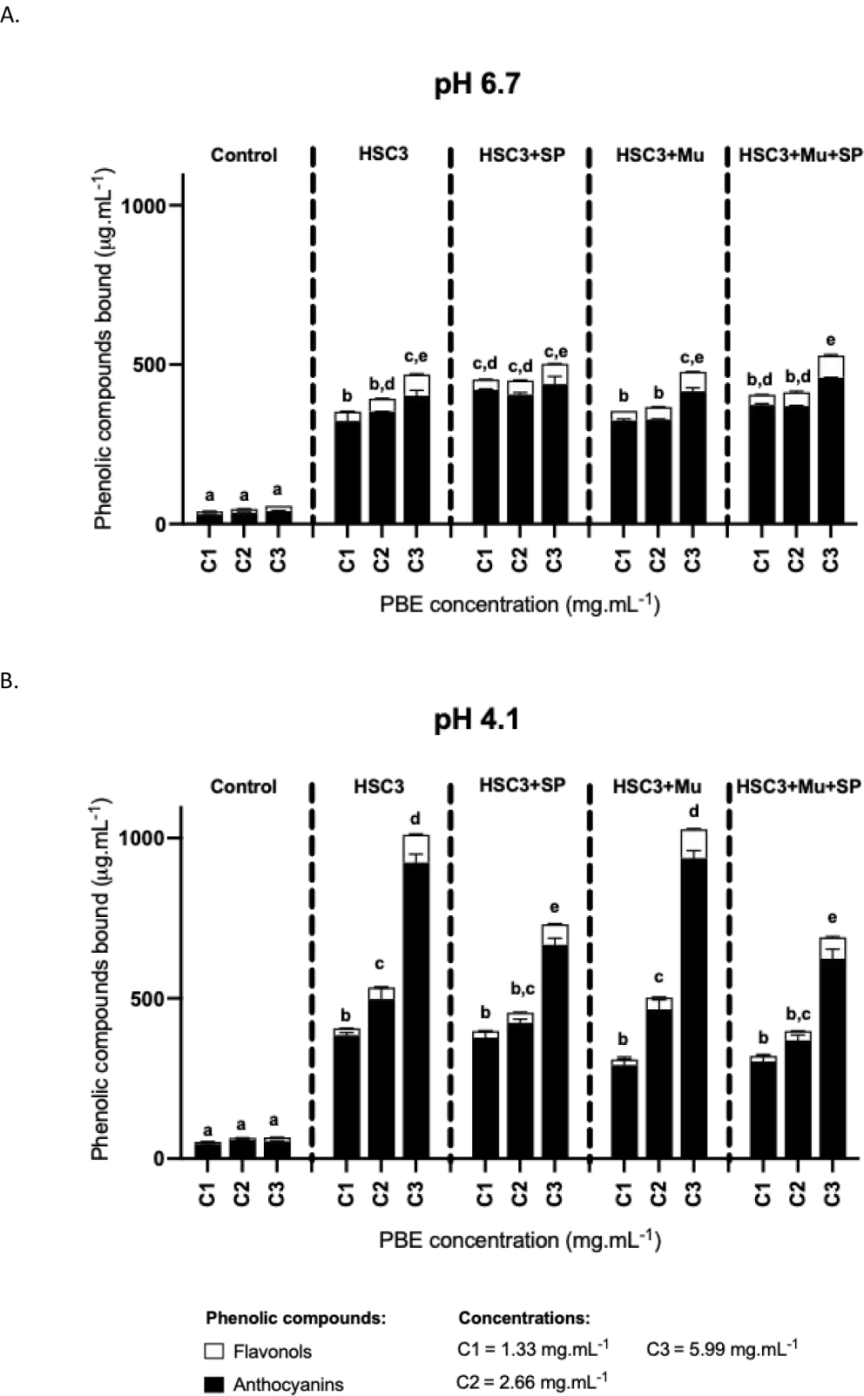
Concentration of the total (bound) phenolic compounds retained in each oral model studied—HSC3 monolayer, HSC3 cells incubated with human saliva (HSC3 + SP), HSC3 cells preincubated with mucin (HSC3 + Mu), and HSC3 cells with both mucosal pellicle and saliva (HSC3 + Mu + SP)—at pH 6.7 (A) and pH 4.1 (B). Concentrations are expressed in equivalents of kaempferol-3-*O*-rutinoside for the flavonols and in equivalents of ternatin D1 for the anthocyanins. Bars represent the contribution of each phenolic family to the total bound concentrations. Data are shown as mean and SEM values for at least three independent experiments; distinct letters on the bars (a, b, c, d, e) indicate statistically significant differences (*p* < 0.05).

At both pH levels, the retention of flavonols (expressed as equivalents of kaempferol-3-*O*-rutinoside) was significantly lower than that of anthocyanins (expressed as equivalents of ternatin D1). This difference can be attributed to the type of phenolic compound rather than to the higher concentration of anthocyanins. Indeed, when the interaction was analyzed in percentage terms, removing the concentration effect (Figure S4), anthocyanins remained the most bound family. Phenolic compound binding to oral components increased with rising concentrations at both pH levels, becoming statistically significant at the highest concentration tested (5.99 mg·mL^–1^). At pH 6.7 (Figure 3A), no quantitative differences among the oral models were observed at this concentration. However, at pH 4.1 (Figure 3B), differences emerged, particularly between models containing salivary proteins and those with only HSC3 cells or mucin (HSC3 + Mu). These findings align with the work of Torres-Rochera et al. (2023), who reported that at wine pH (pH 3.6), the anthocyanin malvidin 3-*O*-glucoside showed a high affinity for mucins (high-molecular-weight salivary proteins), driven by hydrophobic interactions and hydrogen bonding.^53^

In addition, the control models with saliva alone (lacking oral cells) showed no interaction (Figure S2). These findings suggest two key points. The first key point is that the presence of salivary proteins in saliva reduces the binding of phenolic compounds to oral cells at pH 4.1. One possible explanation is the formation of soluble aggregates between the PBE phenolic compounds ((poly)­acylated anthocyanins and flavonols) and salivary proteins, instead of the typical insoluble polyphenol-salivary protein aggregates. This is supported by the SDS-PAGE results (Figure 7), which show that the salivary proteins remaining in solution after interaction with PBE are nearly the same as those in control saliva. Further supporting the hypothesis of the formation of soluble aggregates between the phenolic compounds from PBE and salivary proteins is the ESI-FIA-MS analysis by Ferrer-Gallego and colleagues, which identified new peaks following the interaction between malvidin 3-*O*-glucoside (a common anthocyanin) and acidic proline-rich proteins.^54^ Another key point is the primary role of the oral cells on phenolic compound interactions, specifically for the (poly)­acylated anthocyanins observed here. This aligns with the research conducted in a previous study by Soares et al., which reported that oral cells were the driving oral component for the interaction of common red wine anthocyanins with the oral constituents and that the presence of salivary proteins also appeared to protect oral cells from these interactions.^31^

Within phenolic compounds, anthocyanins are of particular interest due to their ability to exist in different structural forms in aqueous solutions, depending on the pH, which underlines their ability to change color.^22^ For common anthocyanins, the red flavylium cation (AH^+^) predominates at very low pH values (typically pH < 1).^21^ As pH increases, two chemical transformation pathways are triggered: deprotonation (pH 4–8), resulting in blue/violet quinoidal base species (A and A^–^), and hydration (pH 3), forming the colorless hemiketal (B), which can tautomerize into yellow chalcones at pH > 3.^21^ Ternatins, a specific group of acylated anthocyanins, exhibit unique equilibria due to their complex structure. Studies on the color and the absorption spectra of *Clitoria ternatea* anthocyanin extract across a pH range of 0.5–13 have suggested that ternatins primarily exist as the magenta-colored AH^+^ form (λ_max_ = 548 nm) at pH < 3, and between pH 4 and 8, the equilibrium shifts toward the A and A^–^ forms, with the proportion of A decreasing significantly at pH 9–10.^55^ Yellow chalcones become prominent at pH > 8, while hydration is inhibited by acyl groups, which provide steric hindrance through intramolecular copigmentation and self-association.^56^ Based on these findings, the present study also explored the chemical species formed during the interaction of PBE with oral constituents at two different pH levels, using UV–visible spectroscopy and HPLC-DAD to identify the anthocyanin species present under the cell assay conditions (Figure S3).

It is important to note that the presence of different anthocyanin species at varying pH levels is not expected to impact the formation of the above-mentioned anthocyanin-salivary protein soluble aggregates. Consistent with this, Ferrer-Gallego et al. found similar dissociation constants between salivary proline-rich proteins and different malvidin 3-*O*-glucoside species at different pH levels: 1.92 mM for the hemiketal form (pH 3.4) and 1.83 mM for the flavylium cation (pH 1.0).^54^

However, regarding the impact on the interaction with oral cells, it can be hypothesized that at pH 6.7, some ternatins exist in the negatively charged anionic quinoidal base form, which may lead to electrostatic repulsion with the negatively charged oral epithelial cell membranes. This repulsion is supported by recent in-house findings showing that giant plasma membrane vesicles (GPMVs) derived from the HSC3 cell line exhibit a negative zeta potential on their surface. Regarding flavonols, pH can also affect their interaction with oral cells. Reported p*K*_a_ values for some flavonols present in this extract (e.g., kaempferol) fall between p*K*_a_ 6 and 7,^57^ suggesting that at pH 6.7, partial deprotonation may occur, resulting in repulsive forces and lower interaction with negatively charged surfaces. These factors may collectively contribute to the lower interaction observed at this pH. On the other hand, the positive charge of the flavylium cation at pH 4.1 can enhance the electrostatic interaction with the oral constituents, promoting stronger binding and complex formation. However, the proportion of flavylium cation seems to be low compared with the neutral quinoidal base form. This neutral form, being more hydrophobic, may have a greater affinity for the phospholipid bilayer of oral epithelial cells, facilitating the interaction with hydrophobic regions. Additionally, the quinoidal base may interact with cell surface proteins, such as mucins, through hydrophobic interactions or hydrogen bonding, further contributing to its binding.

In summary, the obtained data can support the hypothesis that anthocyanins from butterfly pea flower tea can interact with constituents within the oral cavity, namely, tongue epithelial cells, and that these interactions could contribute to astringency development. The presence of salivary proteins appears to significantly decrease these interactions. At pH 4.1, the interactions are primarily driven by the neutral quinoidal base form of ternatins associated with oral epithelial cell membranes, along with electrostatic interactions involving the flavylium cation and oral constituents, although to a lesser extent. In contrast, at pH 6.7, electrostatic repulsion may occur between the anionic quinoidal base form of ternatins and negatively charged oral epithelial cell membranes. While the anthocyanin group plays a major role in these interactions, the potential influence of flavonols cannot be ruled out. Although this oral model mimics, to its best capacity, the dynamic and complex oral environment and integrates key components of the oral cavity, it cannot account for other mechanisms occurring in the mouth during food consumption, which can affect the astringency perception. Factors such as mechanical forces from chewing, continuous saliva flow, and the presence of diverse oral microbiota are not considered, potentially affecting the interaction dynamics. Nonetheless, previous studies have consistently shown that phenolic compounds can interact with oral epithelial cells.^15,43,58,59^ The interaction with these cells may contribute to astringency development in two distinct ways: indirectly, as the main structural component of the oral mucosa onto which the mucosal pellicle is formed, and directly, by disruption of the oral mucosal pellicle during food and beverage intake and exposure of the underlying oral epithelium, allowing (direct) interactions of astringent compounds to it. The importance of this thin layer in astringency perception has been highlighted by Nayak and Carpente.^58^ Moreover, Reis and colleagues provided insights into how polyphenols interact with model membranes mimicking oral epithelial surfaces, suggesting that such interactions may alter cell membrane properties relevant to sensory perception.^60^ Although sensory data are not available in the present study, the observed interactions between anthocyanins from butterfly pea flower tea and human epithelial cells align with these mechanistic insights and provide a plausible molecular basis for their contribution to the perception of astringency.

### Interaction of the Individual Phenolic Compounds: Influence of the Oral Components and Structure of Phenolic Compounds

After identifying the conditions exhibiting the most significant overall interactions (Figure 3), an HPLC analysis was performed to identify which phenolic compounds in the mixture were mainly bound by the oral model (see section HPLC Analysis of the Interactions of the PBE with the Oral Model in the Experimental Section). Since oral epithelial cells are the main drivers of these interactions, subsequent analyses focused on the HSC3 cell-only model. Figure 4 shows the concentrations of key phenolic compounds from each family retained by the HSC3 model at pH 6.7 (Figure 4A) and pH 4.1 (Figure 4B), representing the conditions of the lowest and highest interactions, respectively. Compounds bound at very low concentrations (below 10 mg·mL^–1^ for the anthocyanins and 1 mg·mL^–1^ for the flavonols family) across the three tested concentrations (1.33, 2.66, and 5.99 mg·mL^–1^) were excluded from this analysis as irrelevant.

**Figure 4:**
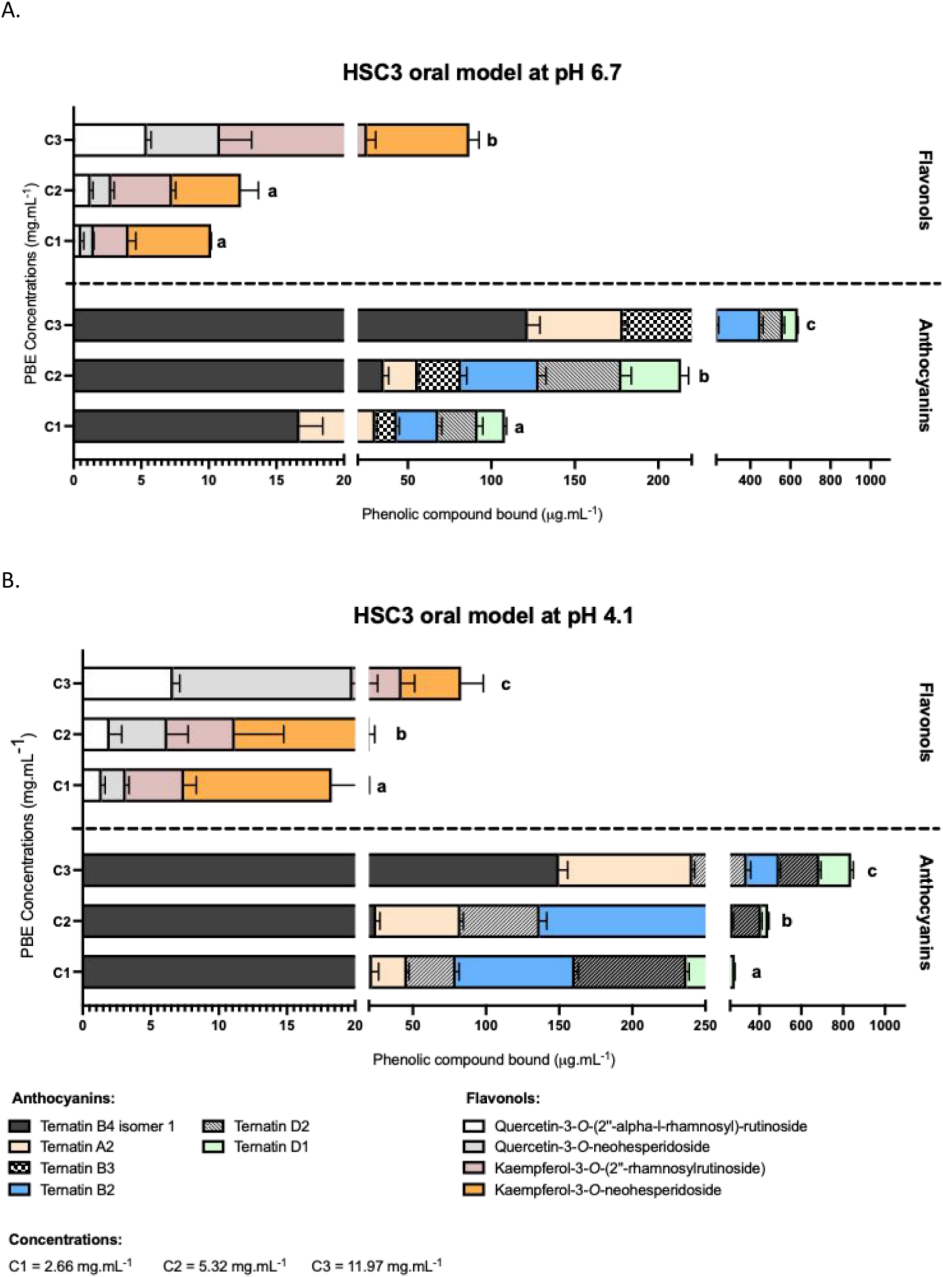
Concentration of the major individual phenolic compounds from each phenolic family retained in the HSC3 oral model at pH 6.7 (A) and at pH 4.1 (B). Data were obtained by HPLC analysis and are expressed as equivalents of kaempferol-3-*O*-rutinoside for the flavonols and ternatin D1 equivalents for the anthocyanins. Results are shown as stacked bar charts representing the mean and SEM values for at least three independent experiments; Different letters on the bars (a, b, c) indicate statistically significant differences (*p* < 0.05).

The anthocyanin group exhibited the highest levels of binding among the phenolic compounds. At mid- and high-range PBE concentrations, these interactions were primarily driven by ternatin B4 isomer 1, ternatin A2, and ternatin B2. Within the flavonol family, although their total retained concentrations were lower (Figure 3), specific compounds displayed notable behavior. Among the flavonols present, kaempferol 3-*O*-neohesperidoside and quercetin 3-*O*-neohesperidoside showed an increase in bound concentration at the highest PBE concentration.

To account for structure-interaction-dependent effects, normalization was applied by considering the relative abundance of each phenolic compound at different concentrations (Figure S4). This approach ensured that binding comparisons were independent of the initial compound concentration. Notably, analysis of the percentage of bound compounds revealed the involvement of additional phenolic compounds in the interactions: ternatin B4 isomer 2 among the anthocyanins and quercetin-3-*O*-rutinoside among the flavonols. In fact, based on their retention percentage, ternatin B4 isomers 1 and 2 were consistently among the most bound anthocyanins. Within the flavonols, a significant increase in retention percentage was observed only at the highest PBE concentration and at pH 4.1, with kaempferol-3-*O*-(2″-O-alpha-rhamnosyl-6″-*O*-malonyl)-beta-glucoside and quercetin-3-*O*-rutinoside exhibiting higher retention percentage levels under these conditions. In general, the binding of each phenolic compound increased at pH 4.1.

It is worth noting that previous studies have shown that quercetin’s additional hydroxyl group enhances its affinity for lipid bilayers compared to kaempferol.^61^ However, this was not observed in this study, likely due to differences in the composition of lipid bilayer models and cell membranes. Furthermore, quercetin has been reported to form complexes with salivary proteins, contributing to heightened perceptions of astringency and bitterness.^62^

In summary, anthocyanins were the primary contributors to the total bound phenolic compound at both pH values and were the most affected by changes in the PBE concentration. Within the anthocyanins group, ternatin B4 isomer 1, ternatin A2, and ternatin B2 were the main contributors to the overall bound concentration of anthocyanins. For flavonols, kaempferol 3-*O*-neohesperidoside showed an increased bound concentration at the higher PBE concentrations.

### Structure–Interaction Relationship of Phenolic Compounds with Oral Constituents

After the above analysis, the bound concentrations (mol·L^–1^) were used alongside structural features of phenolic compounds, including molecular weight, number of hydroxyl groups, and the presence of malonyl, ρ-coumaroyl, d-glucosyl and rhamnosyl residues, to perform principal component analysis (PCA) and Pearson correlation analysis. These statistical analyses allowed for a clearer visualization of the data and facilitated the identification of the extent to which each characteristic (variable) influenced the interaction with the oral model (HSC3). Figures 5 and 6 present the PCA and the Pearson correlation data for the oral model (HSC3) that exhibited the highest interaction for anthocyanins and flavonols present in the PBE, at pH 6.7 and 4.1, respectively.

**Figure 5:**
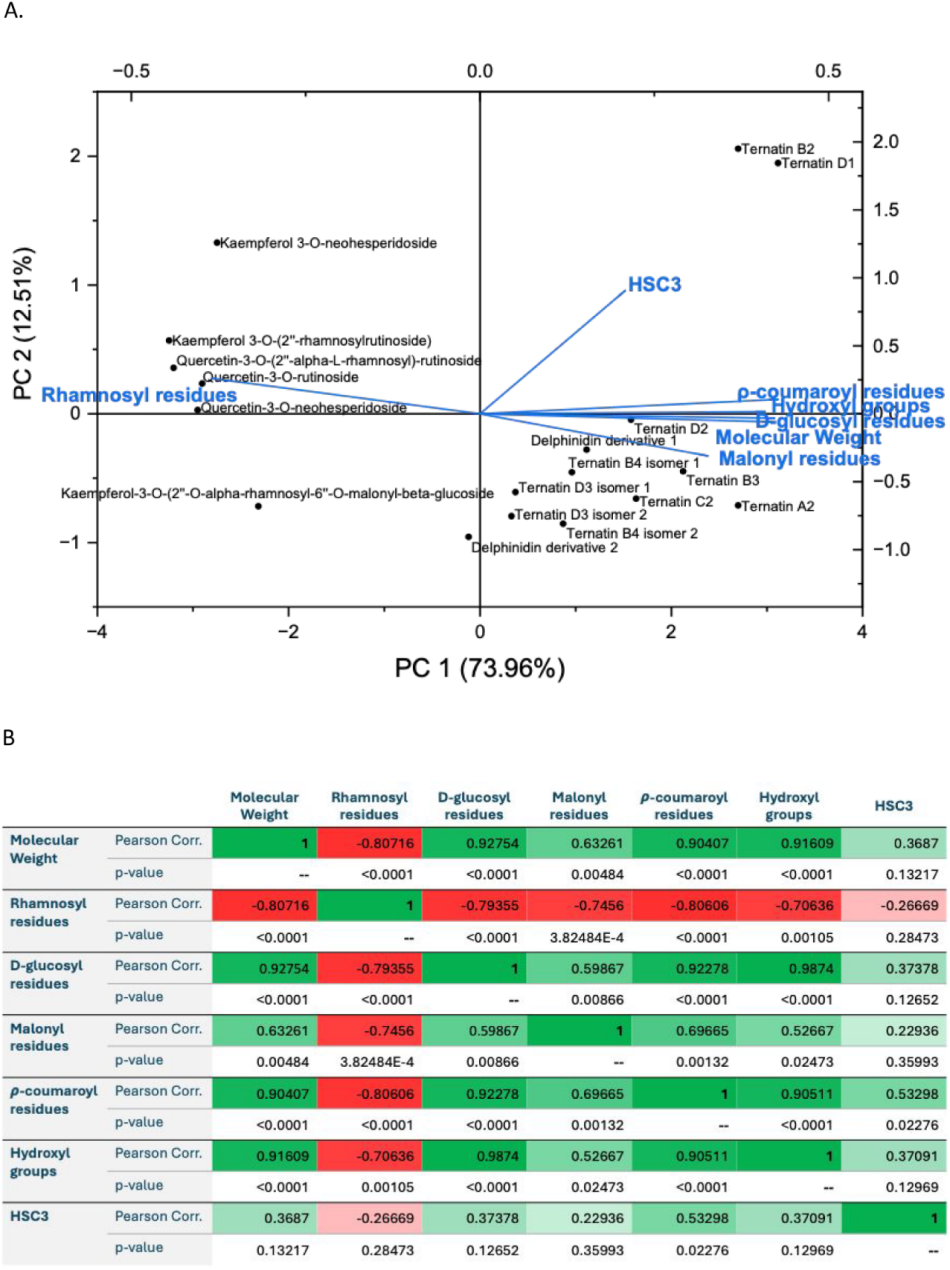
PCA data (A) and Pearson analysis (B) for the oral model (HSC3) that exhibited the highest interaction for the different phenolic compounds present in the PBE at pH 6.7. This analysis utilized the bound concentrations (mol·L^–1^) data set alongside phenolic compound structure parameters, including molecular weight, number of hydroxyl groups, and the presence of malonyl, ρ-coumaroyl, d-glucosyl, and rhamnosyl residues.

**Figure 6:**
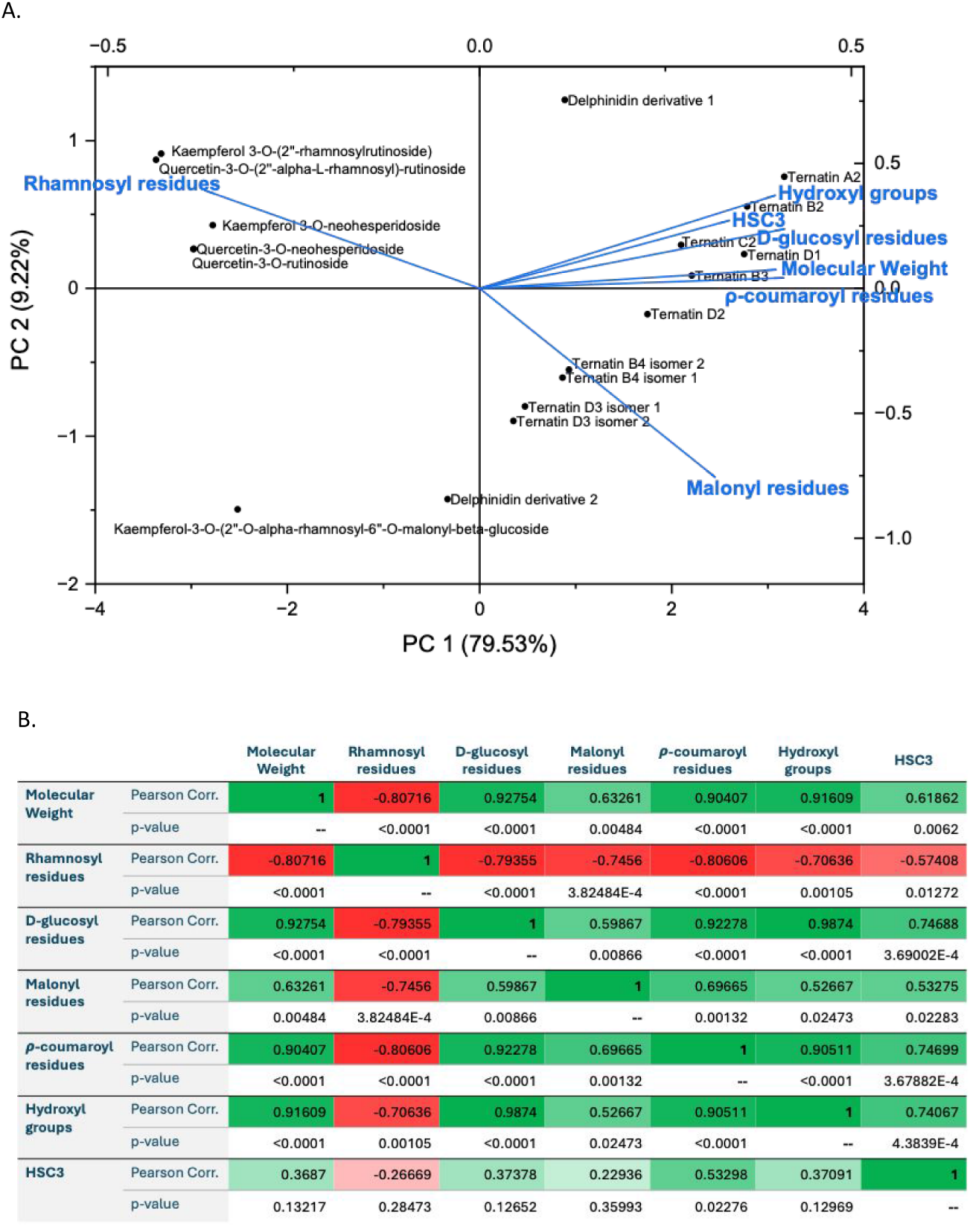
PCA data (A) and Pearson analysis (B) for the oral model (HSC3) that exhibited the highest interaction for the different phenolic compounds present in the PBE at pH 4.1. This analysis utilized the bound concentrations (mol·L^–1^) data set alongside phenolic compound structure parameters, including molecular weight, number of hydroxyl groups, and the presence of malonyl, ρ-coumaroyl, d-glucosyl, and rhamnosyl residues.

In Figure 5A, the PCA performed at pH 6.7 shows that the first component (PC 1) accounts for 73.96% of the variance, while the second component (PC 2) explains 12.51%. Together, they capture 86.47% of the total variability in the behavior of the phenolic compounds found in PBE. Analysis of the extracted eigenvectors reveals that PC 1 is strongly associated with four structural features: the molecular weight of each compound (loading = 0.418), the number of d-glucosyl (0.422), *p*-coumaroyl (0.427), and hydroxyl groups (0.409) within the phenolic compound. These positive loadings suggest that these features tend to co-occur. Conversely, the number of rhamnosyl residues present in the phenolic compound shows a negative loading of −0.384 in PC 1, indicating an inverse relationship to the positively loaded features. PC 2 is primarily influenced by the HSC3 oral model, which exhibits a strong positive loading of 0.906, indicating that this variable drives variation along this component.

In addition, the spatial positioning of the phenolic compounds in the biplot reflects their association with these variables. For example, ternatin D2 and delphinidin derivative 1 appear on the right side of the biplot along PC 1, suggesting a strong association with positively loaded variables such as *p*-coumaroyl, hydroxyl, d-glucosyl, and malonyl residues as well as higher molecular weight. Other compounds, including ternatin B3, ternatin A2, ternatin C2, ternatin B4 isomers and ternatin D3 isomers, also cluster on the right side, mainly associated with the presence of malonyl residues. In contrast, ternatin B2 and ternatin D1 are positioned higher along PC 2, indicating a greater influence from the HSC3 oral model. On the opposite (left) side of the biplot is located the rhamnosyl residues parameter, which has an inverse relationship with the positively loaded features in PC 1. Compounds like kaempferol and quercetin are located nearby, suggesting they are more influenced by this structural feature. In summary, the PCA biplot analysis highlights a clear contrast between anthocyanins and flavonols, as they are positioned on opposite sides of the PCA plot. Anthocyanins cluster with structural features that load positively on PC 1, namely, molecular weight, and the number of d-glucosyl, *p*-coumaroyl residues, and hydroxyl groups. In contrast, flavonols are more associated with features negatively loaded on PC 1, particularly the presence of rhamnose residues.

The Pearson correlation analysis (Figure 5B) further complements the PCA findings by analyzing the direct linear relationship between the variables. In particular, the HSC3 oral model shows moderate positive correlations with the presence of ρ-coumaroyl residues (0.53298). Other structural parameters, such as d-glucosyl residues (0.37378), hydroxyl groups (0.37091), malonyl residues (0.22936), and the molecular weight of the phenolic compounds (0.3687), also exhibited positive correlations with the HSC3 oral model, although these were not statistically significant (*p*-value >0.05). These results align with the PCA results, as these variables account for a significant portion of the variability in the data set, although they do not have higher loadings in PC 2, where the HSC3 oral model is the main driver. Hence, these findings suggest that phenolic compounds with higher quantities of ρ-coumaroyl may result in stronger interactions with the oral epithelial cells.

The PCA at pH 4.1 (Figure 6A) reveals that PC 1 accounts for 79.53% of the variance in the data, while PC 2 explains 9.22%, together capturing 88.75% of the total variability in the behavior of the phenolic compounds found in PBE. Compared to the results at pH 6.7, PC 1 explains a larger portion of the variation at pH 4.1, while PC 2 contributes slightly less. The extracted eigenvectors show that PC 1 remains primarily influenced by the same structural features observed at pH 6.7: molecular weight of each compound (loading = 0.398), number of d-glucosyl (0.410), ρ-coumaroyl (0.409), and hydroxyl groups (0.39735). However, the contributions of these parameters are slightly reduced, except for the HSC3 oral model, whose loading increases from 0.20794 at pH 6.7 to 0.335 at pH 4.1, indicating a more prominent role under acidic conditions. For PC 2, a shift is also observed. The loading for rhamnosyl residues increases to 0.392, while the influence of the HSC3 oral model decreases substantially from 0.906 at pH 6.7 to 0.272 at pH 4.1. This reduction suggests that HSC3 contributes less to the variation captured by PC 2 at lower pH. The biplot illustrates that flavonols continue to cluster with structural parameters, such as the number of rhamnosyl residues. Structural features such as the number of d-glucosyl, hydroxyl, and *p*-coumaroyl residues in each compound, along with their molecular weight, continue to group together and now appear spatially closer to HSC3 at pH 4.1, indicating a stronger covariation. Regarding the proximity of the phenolic compound to specific structural parameters or the oral model, anthocyanins also shift positions: ternatin D1 and ternatin B2, which were previously closely associated with HSC3 at pH 6.7, now align more with structural parameters like hydroxyl and d-glucosyl residues and their molecular weight. Similarly, ternatin A2, ternatin B3, and ternatin C2, which were positioned near the malonyl residue parameters at pH 6.7, are now located near the HSC3 oral model parameters and structural parameters. This suggests an increased influence of the HSC3 oral model on these compounds under acidic conditions. Overall, the PCA results indicate stronger covariation between anthocyanin structure and the HSC3 oral model at pH 4.1, likely reflecting changes in molecular interactions or compound stability under acidic conditions.

The results from the Pearson correlation analysis at pH 4.1 (Figure 6B) were consistent with those at pH 6.7, with the number of ρ-coumaroyl residues showing the strongest positive correlation with the HSC3 model (+0.74699). However, at 4.1, additional structural features also exhibited statistically significant positive correlations with the HSC3 oral model, namely, the number of d-glucosyl residues (+0.74688), hydroxyl groups (+0.74067), malonyl residues (+0.53275), and molecular weight (+0.61862). In contrast, the number of rhamnosyl residues displayed a significant negative correlation (−0.57408), suggesting that this moiety may hinder interactions with the HSC3 oral model under acidic conditions. Notably, this trend contrasts with the findings of Scharbert and colleagues, who reported that the addition of a rhamnosyl moiety (specifically to quercetin) lowered the astringency threshold of flavonols, whereas a glucose attached directly to the aglycone increased it.^63^ While the present study did not include sensory evaluation of the individual compounds, the strong positive correlation between d-glucosyl moieties and HSC3 interactions, together with the negative correlation for rhamnosyl residues, suggests that d-glucosyl groups may enhance and rhamnosyl groups may limit oral interactions linked to astringency under acidic conditions. To confirm the role of rhamnosyl residues in the binding of phenolic compounds to oral constituents, an additional experiment was conducted under acidic conditions (pH 4.1) using the same cell-based setup described herein. An equimolar mixture (250 μmol·L^–1^) of four flavonol compounds was tested: quercetin-3-*O*-rutinoside (rutin), kaempferol-3-*O*-rutinoside, and their respective aglycones. The results showed that both flavonols containing rhamnosyl residues exhibited significantly lower adsorption compared with their aglycones in both the HSC3 and HSC3 + Mu + SP oral models (Figure S5). This supports the hypothesis that rhamnosyl residues limit phenolic binding and may reduce astringency-related interactions. Furthermore, upon comparison of the adsorption of quercetin-3-*O*-rutinoside in the PBE matrix to that in the equimolar mixture, adsorption was significantly higher in the PBE, suggesting that the matrix may enhance interactions, potentially through synergistic effects with other compounds. This enhanced adsorption may result from noncovalent synergistic interactions, including copigmentation phenomena, which are well documented in polyacylated anthocyanins, such as ternatins.^64^ They are known to undergo intramolecular and/or intermolecular stacking interactions between the chromophore and aromatic acyl groups on their sugar moieties.^64^ These conformational arrangements stabilize the quinoidal base form of anthocyanins in aqueous environments near neutral pH, contributing to their intense blue coloration.^65^ Such structural arrangements may also increase molecular stability and potentially enhance the binding affinity to biological surfaces. Therefore, it is plausible that copigmentation phenomena, either intramolecular or involving other flavonoids in the PBE, such as quercetin or kaempferol, may contribute to the enhanced adsorption observed in the PBE matrix compared to isolated flavonols, highlighting the relevance of considering matrix effect, such as flavonoid–flavonoid interactions, in astringency-related studies.

In summary, phenolic compounds with a higher *p*-coumaroyl residue content exhibit stronger interactions with oral epithelial cells. Acidic conditions appear to amplify these interactions,^66^ especially for anthocyanins rich in d-glucosyl and hydroxyl groups.^59^

### Salivary Proteins Involved on the Interaction with Phenolic Compounds in the Different Oral Models

The effect of the above-mentioned interactions on the salivary protein profile was also studied. For this purpose, SDS-PAGE analysis was performed on the supernatants obtained from the HSC3 + Mu + SP model at pH 4.1, focusing on the lowest (1.33 mg·mL^–1^) and highest (5.99 mg·mL^–1^) PBE concentrations, conditions under which significant interactions were observed. This analysis aimed to provide insights into the specific salivary proteins actively involved in interactions with the PBE. The SDS-PAGE results, presented in Figure 7, show that increasing PBE concentrations led to a decrease in the intensity of specific protein bands in the supernatants. This observation suggests that certain salivary proteins, characterized by their reduced band intensity following incubation with PBE, were precipitated as a result of their interactions with the phenolic compounds present in the extract.

**Figure 7:**
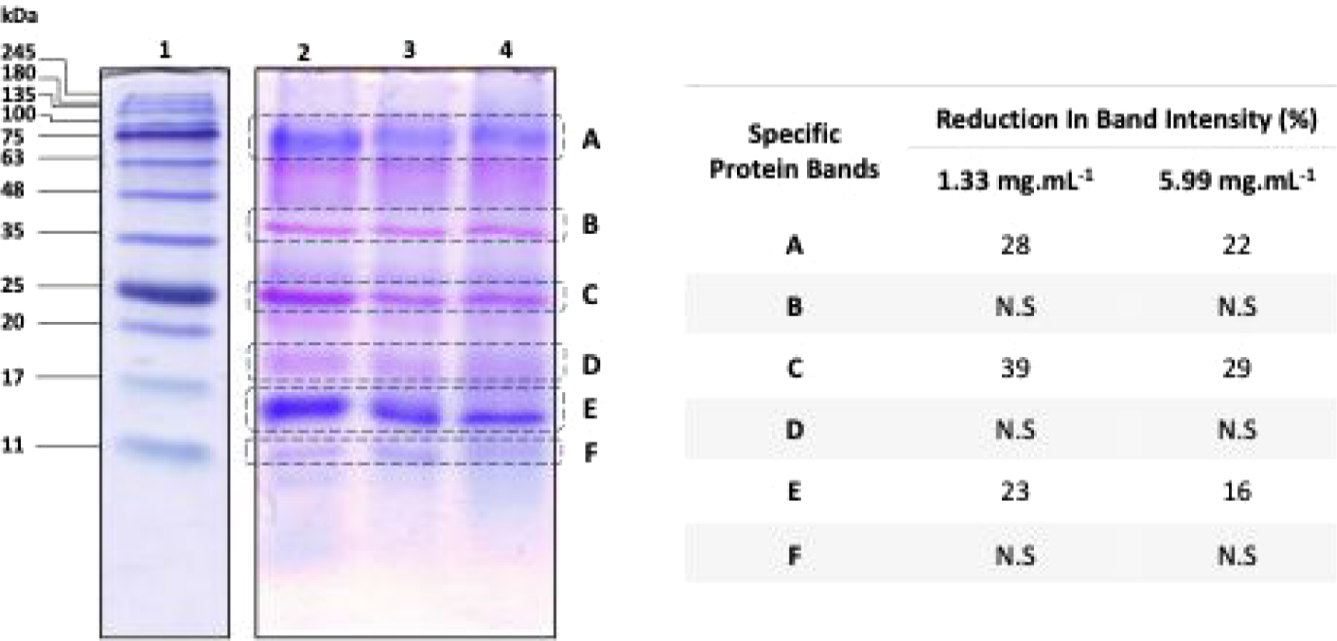
Electrophoresis analysis (left) (SDS-PAGE, 16%) of the supernatant of saliva control (lane 2) as well as the supernatants of the interactions of PBE with the HSC3 + Mu + SP model at pH 4.1 at 1.33 mg·mL^–1^ (lane 3) and at 5.99 mg·mL^–1^ (lane 4). The protein marker (lane 1) has the molecular weight markers highlighted with lines and the molecular mass expressed in kDa. The table (right) indicates the values of the percentage decrease in the specific protein band densities after the interactions of PBE with the HSC3 + Mu + SP model at pH 4.1 at 1.33 mg·mL^–1^ and at 5.99 mg·mL^–1^ (right). N.S. indicates “Not Significant”. Specific protein bands are labeled as follows: A (glycosylated salivary alpha-amylase), B (basic PRP), C (acidic PRP), D (PRP), E (cystatin S), and F (cystatin B).

In Figure 7, six distinct bands can be identified. Four of these bands (Bands B–D) display a pink-violet staining pattern, while the remaining three take on a blue color (Bands A, E, and F). This pinkish coloration, well documented by previous researchers, is a recognized characteristic of salivary proline-rich proteins (PRPs).^6,67,68^ This staining pattern results from the use of R250 Coomassie Blue staining solution and a destaining solution lacking organic solvents, particularly because of the destaining step.^67,68^ It is important to highlight that PRPs, due to their lack of a well-defined protein structure and unusually high proline content, exhibit altered migration patterns on SDS-PAGE gels. Their rigidity and reduced affinity to bind to SDS result in slower migration compared to globular proteins of similar molecular weight, leading to an apparent molecular weight that is typically 1.2 to 1.8 times higher than their actual molecular weight.^68^ Regarding band A, its size intensity and location around 62 kDa suggest it corresponds to glycosylated salivary alpha amylase.^69^ This band showed decreases of 28% and 22% (Figure 7) at the lowest and highest PBE concentration, respectively. Band B, located around 35 kDa, is identified as a basic PRP. This identification aligns observations by Beeley et al. (1991) at a similar region (37 kDa),^67^ and with ESI mass analysis from Soares et al. (2011),^6^ who identified basic PRPs with a molecular weight of 23.4 kDa, corresponding to an apparent molecular weight of 35.1 kDa when adjusted with a 1.5 multiplication factor.^6^ This protein did not exhibit a measurable interaction with PBE. Band C, at approximately 25 kDa and displaying a distinct pink-violet coloration, is identified as an acidic PRP. Although Beeley et al. (1991) did not specify the PRP subtype,^67^ Soares et al. (2011) identified it as an acidic PRP with an apparent molecular weight range of 24–29 kDa and an ESI mass analysis of 15.5 kDa.^6^ When applying their adjustment factor of 1.8, the weight aligns at 27.9 kDa, but using a factor of 1.5 aligns it with our results at 23.25 kDa.^6^ This acidic PRP exhibited significant interaction with PBE, with decreases of 39% and 29% at the lowest and highest concentrations, respectively. Band D, located above 17 kDa, also displayed a pink-violet coloration, indicating that it is likely a PRP, although the subtype could not be determined from the available literature. This band did not show measurable interaction with PBE. Finally, the lowest molecular weight bands (Bands E and F) are identified as cystatin S and cystatin B, respectively.^70^ Band E exhibited decreases of 23% and 16% upon PBE interaction at the lowest and highest concentrations, respectively, while Band F did not display significant changes.

In summary, the SDS-PAGE analysis provided clear evidence of the involvement of PRPs and other salivary proteins in interactions with phenolic compounds, particularly evident in the marked reduction of band intensities corresponding to glycosylated salivary alpha-amylase (Band A), acidic PRP (Band C), and cystatin S (Band E). While previous studies have documented interactions between PRPs (including glycosylated basic PRP and acidic PRP) with flavonols and anthocyanins,^20,31^ the specific interaction of cystatins with these phenolic compounds represents a novel finding. Although earlier reports suggested the potential for such interactions, particularly with polyphenol oxidation products like CQA dehydrodimers,^71^ this study provides the first detailed evidence of direct interactions between cystatins and phenolic compounds. Furthermore, as discussed by Li et al. (2021), the structural characteristics of anthocyanins, such as their hydroxyl groups and conjugated double bonds, likely play a crucial role in facilitating protein binding, underscoring their potential for interaction with cystatins.^72^

This work sheds light on how phenolic compounds present in a typically astringent matrix, such as *C. ternatea* tea, interact with oral components, offering mechanistic insights into molecular interactions that may underlie the perception of astringency. Focusing on two families of phenolic compounds, anthocyanins and flavonols, at pH 6.7 and 4.1, the study identified anthocyanins, particularly those with higher *p*-coumaroyl content, as the primary contributors to oral interactions, displaying binding to oral constituents stronger than that of flavonols. Oral epithelial cells were shown to play a significant role in these interactions, while salivary proteins, especially amylase, acidic PRPs, and cystatins, modulated these interactions to varying extents. The acidic environment enhanced the binding capacity of phenolic compounds, with the neutral quinoidal base form of anthocyanins driving interactions with epithelial membranes, while the flavylium cation contributed more modestly via electrostatic forces. Notably, while pH influenced the binding strength, it did not alter the overall interaction potential of the different phenolic compounds with oral components.

Although the oral cell-based model employed in this study offered valuable insights, it presents inherent limitations. The model consists of a monolayer of epithelial cells, lacking the multilayered architecture of the oral mucosa and the dynamic flow of saliva, both of which may affect the binding of phenolic compounds. Additionally, it does not replicate mechanical forces, such as tongue movement and mastication, which can further impact phenolic compound interactions and perception.

Beyond these model limitations, predicting phenolic compound interactions in complex matrices such as PBE requires more than general chemical descriptors. Further systematic studies using phenolic mixtures are necessary to clarify the structural determinants of these interactions. Another important avenue for future research is the isolation of the different anthocyanins, particularly those with high interaction potential and the establishment of dose–response thresholds (DoT values), which are currently lacking.

In conclusion, this study enhances our understanding of the astringency mechanisms in beverages. It highlights the roles of distinct families of phenolic compounds (anthocyanins and flavonols), oral constituents, and pH in shaping the mechanisms of astringency perception. These findings provide valuable insights for product development and sensory optimization in the food industry. From a practical perspective, selecting phenolic compounds with higher rhamnosyl residues or lower *p*-coumaroyl residues may help reduce perceived astringency. Additionally, formulating beverages at neutral pH could be an effective strategy to modulate astringency intensity. Future investigations should include sensory validation of these interactions and explore how optimizing the phenolic compound concentration and pH in anthocyanin-rich beverage formulations can fine-tune the perception of astringency. Moreover, the potential impact of other beverage compounds, such as polysaccharides, on sensory attributes like mouthfeel and aftertaste should also be investigated to support the design of consumer-preferred formulations.

## Supplementary Material



## Notes

Viability (%) of cells, Concentration of the total (bound) phenolic compounds retained in control saliva, UV–visible spectrum of the PBE at pH 4.1 and 6.7, Total percentage of interaction for anthocyanins and flavonols at pH 4.1 and 6.7 in PBE matrix, Total percentage of interaction for flavonols at pH 4.1 in a equimolar mixture (PDF)

## Abbreviations

PRPs: proline-rich proteins
SDS-PAGE: sodium dodecyl sulfate–polyacrylamide gel electrophoresis
TFA: trifluoroacetic acid
HPLC: high-performance liquid chromatography
BSA: bovine serum albumin
DTT: dithiothreitol
SDS: sodium dodecyl sulfate
TEMED: *N*,*N*,*N*′,*N*′-tetramethylethylenediamine
TCA: trichloroacetic acid
PBE: phenolic compounds-rich butterfly pea extract
LC-MS: Liquid Chromatography–Mass Spectrometry
PBS: Phosphate Buffered Saline
DMSO: Dimethyl sulfoxide
PCA: Principal component analysis
