# Supplementary material for: Molecular Insights into the Astringency of Clitoria ternatea Tea: Role of Phenolic Structure, Oral Constituents, and pH
Source: J Agric Food Chem. 2025 Oct 10;73(42):26957–71. doi: 10.1021/acs.jafc.5c06189 (PMC12818758; doi:10.1021/acs.jafc.5c06189)
Supplement: Supplementary file 1 [file jf5c06189_si_001.pdf]

Supporting information

Molecular insights into the astringency of Clitoria ternatea tea: role of phenolic structure, oral constituents, and pH

Inês E. Silva, Joana Vieira, Carlos Guerreiro, Joana Oliveira, Elsa Brandão, Victor de Freitas, Susana Soares*

REQUIMTE, LAQV, Department of Chemistry and Biochemistry, Faculty of Sciences,

University of Porto, Rua do Campo Alegre, s/n, 4169-007 Porto, Portugal, *

susana.soares@fc.up.pt.

Cell viability assay

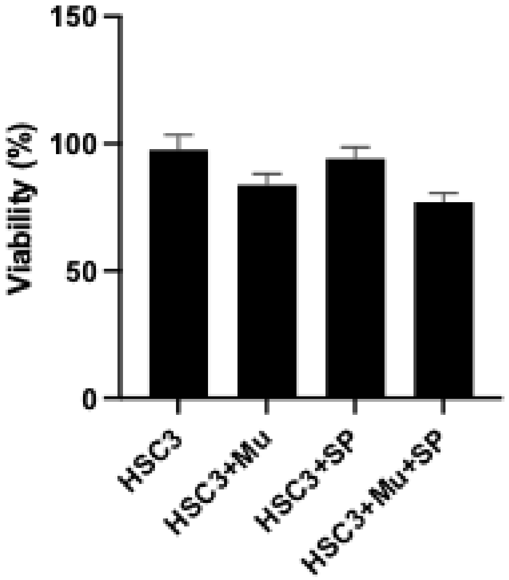

Figure S1: Viability (%) of cells within each condition tested by neutral red assay. No significant statistically differences were observed.

Interaction of the PBE with the oral models

A.

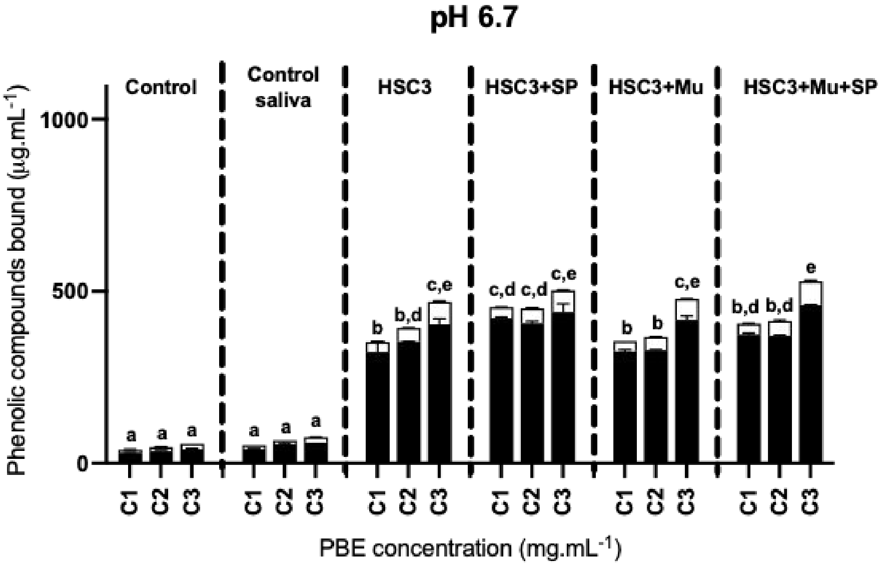

B.

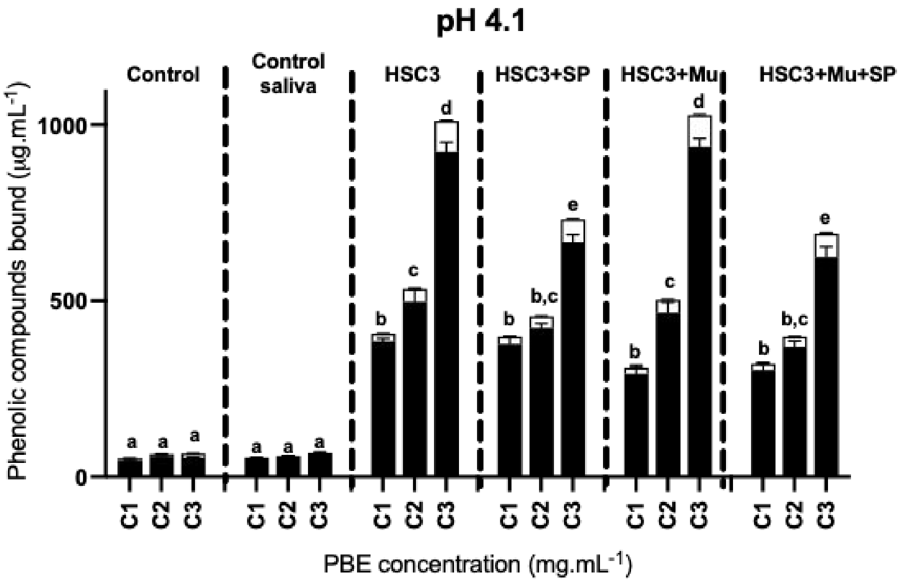

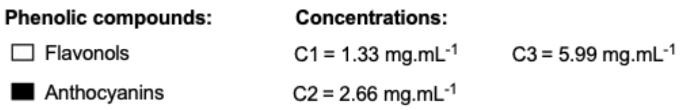

Figure S2: - Concentration of the total (bound) phenolic compounds retained in each oral model studied at pH 6.7 (A) and pH 4.1 (B). Concentrations are expressed in equivalents of kaempferol-3-O-glucoside for the flavonols family and expressed in equivalents of ternatin D1 for the anthocyanin family. Data are presented as bars showing the contribution of each phenolic compound family to the total bound concentrations. Data are presented as the mean and SEM values for at least three independent experiments; distinct letter associations from each bar represent statistically different results ($p<0.05$).

In Figure S3, it can be observed that at pH 4.1, the UV-visible spectrum of the PBE showed three maximum absorption wavelengths at 535, 575 nm, and 618 nm. These features suggest the coexistence of the flavylium cation (λ_{max} = 535 nm) and the neutral quinoidal base (λ_{max} = 575 and 618 nm). At pH 6.7, the shoulder at 535 nm, associated with the flavylium cation, disappears, while the absorption at 618 nm undergoes a bathochromic shift to 622 nm and a hyperchromic effect, indicating the conversion of the neutral quinoidal base into the anionic one. These results concur with the ones observed by Pereira et al. (2024), who studied the pH-dependent color change of a PBE using UV-visible spectroscopy (250-800 nm) and CIELAB color coordinates [31].

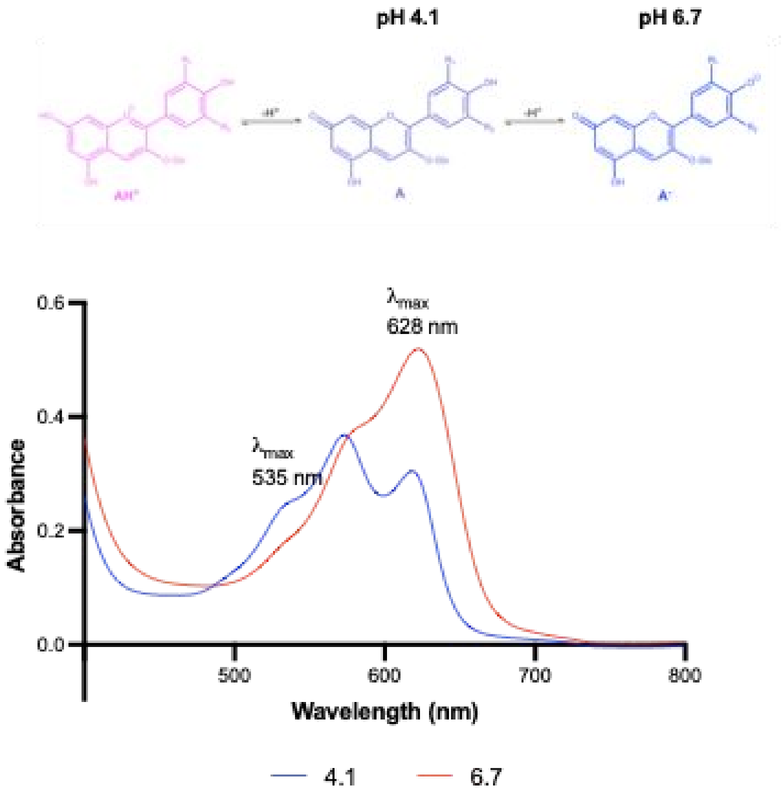

Figure S3: UV-Visible spectra for PBE at the final pH values after interactions with the oral constituents (4.1 and 6.7). The R_1 and R_2 present different substitution patterns that include different numbers of glucose moieties and acyl groups.

Interaction of the individual phenolic compounds: Influence of the oral components and phenolic compound's structure

A. B.

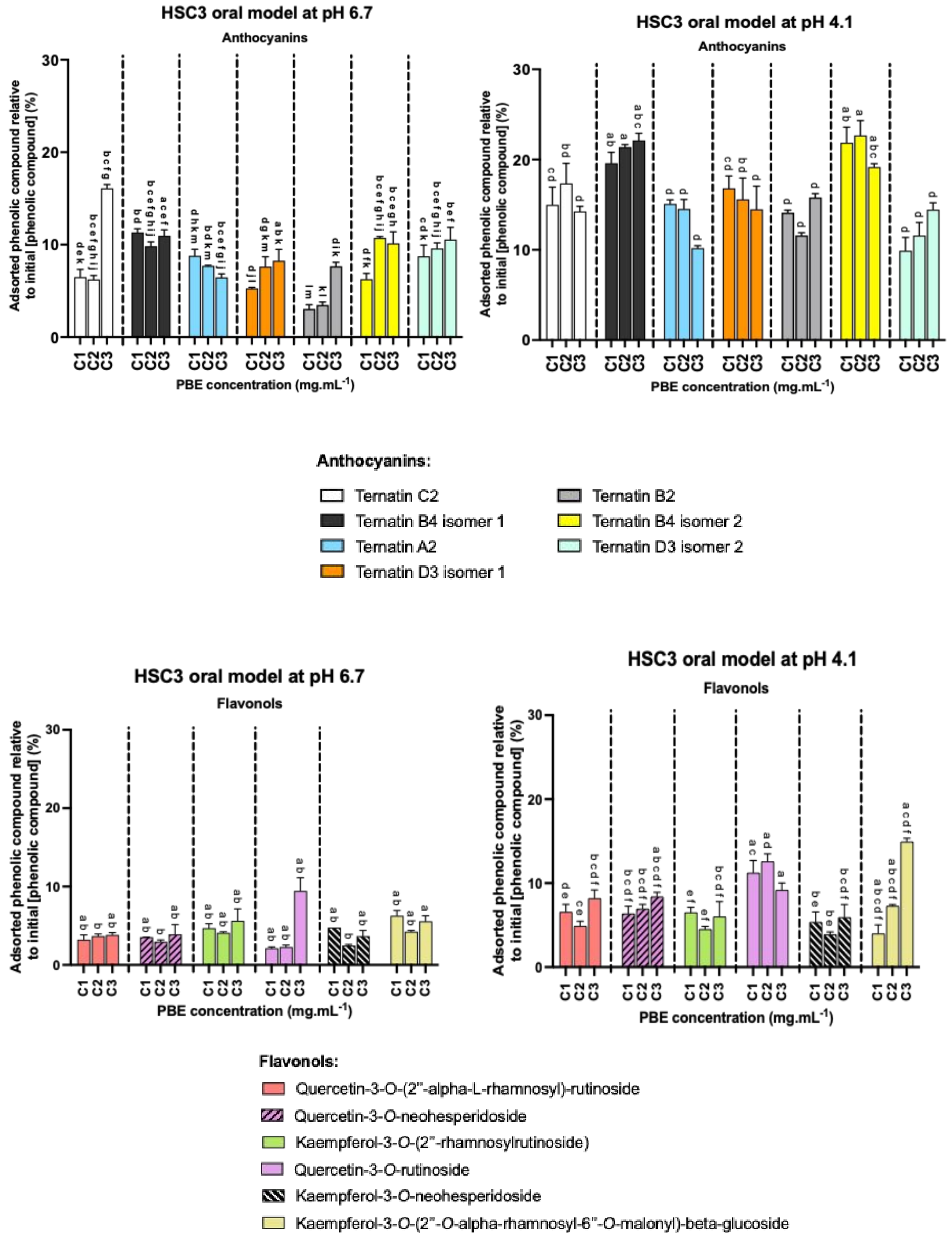

Figure S4: Total percentage of interaction upon concentrations normalization for anthocyanins and flavonols retained by HSC3 oral model at pH 6.7 (A) and pH 4.1 (B). Data are presented as the mean and SEM values for at least three independent experiments; distinct letter associations from each bar represent statistically different results ($p<0.05$).

Interaction of an equimolar mixture of pure phenolic compounds with the oral models

To further examine the influence of rhamnosyl residues on oral interactions, the binding behavior of quercetin-3-*O*-rutinoside, kaempferol-3-*O*-rutinoside, and their respective aglycones was assessed using the HSC3 oral model under acidic conditions (pH 4.1). As shown in Figure S5, both flavonols that contain rhamnosyl residues exhibited significantly lower adsorption compared to their aglycones in both the HSC3 and HSC3+Mu+SP oral models.

Quercetin-3-*O*-rutinoside showed ~1.5% adsorption in both models, while its aglycone reached ~6% adsorption in the HSC3 model and ~3% in the HSC3+Mu+SP model. Kaempferol-3-*O*-rutinoside showed ~5% and ~3% adsorption in the HSC3 and HSC3+Mu+SP model, respectively, while the aglycone showed ~8% and ~6%. These results suggests that the presence of rhamnosyl residues reduces the affinity of these compounds for oral constituents. These results are consistent with the Pearson correlation at pH 4.1 and support the hypothesis that rhamnosyl residues limits phenolic compound binding and may reduce astringency-related interactions.

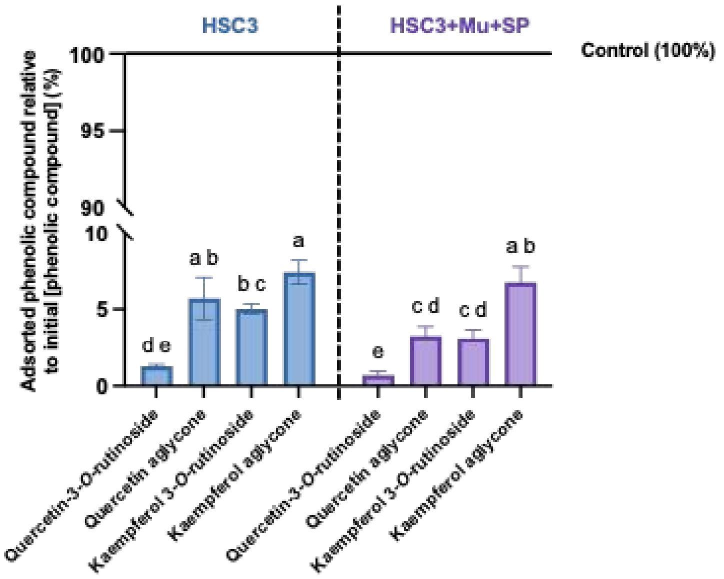

Figure S5: : Total percentage of interaction upon concentrations normalization for flavonols retained by HSC3 and HSC3+Mu+SP oral models at pH 4.1. Data are presented as the mean and SEM values for at least three independent experiments; distinct letter associations from each bar represent statistically different results ($p<0.05$).

Furthermore, when comparing the adsorption of quercetin-3-*O*-rutinoside in the BPE (Figure S4) to that in this equimolar mixture (Figure S5), notable differences were observed. In the PBE, tested at 1.33, 2.66, and 5.97 $mg.mL^{-1}$ (corresponding to 24, 48, and 100 $\mu mol.\ L^{-1}$ of quercetin-3-*O*-rutinoside), adsorption ranged from 7% to 13%. In contrast, when quercetin-3-*O*-rutinoside was tested in a equimolar mixture at 250 µM, adsorption dropped to ~1–2%. This indicates that the PBE matrix seems to enhance quercetin-3-*O*-rutinoside's interaction with the HSC3 oral model, possibly through synergistic effects or interactions with other compounds that facilitate binding. These data reveal the importance of the matrix when studying these types of interactions.
